# A high-latitude fauna of mid-Mesozoic mammals from Yakutia, Russia

**DOI:** 10.1371/journal.pone.0199983

**Published:** 2018-07-25

**Authors:** Alexander Averianov, Thomas Martin, Alexey Lopatin, Pavel Skutschas, Rico Schellhorn, Petr Kolosov, Dmitry Vitenko

**Affiliations:** 1 Zoological Institute of the Russian Academy of Sciences, Saint Petersburg, Russia; 2 Institute of Earth Sciences, Saint Petersburg State University, Saint Petersburg, Russia; 3 Institute of Geology and Petroleum Technology, Kazan Federal University, Kazan, Russia; 4 Steinmann-Institut für Geologie, Mineralogie und Paläontologie, Universität Bonn, Bonn, Germany; 5 Borissiak Paleontological Institute, Russian Academy of Sciences, Moscow, Russia; 6 Vertebrate Zoology Department, Saint Petersburg State University, Saint Petersburg, Russia; 7 Institute of Diamond and Precious Metals Geology, Siberian Branch of the Russian Academy of Sciences, Yakutsk, Russia; Perot Museum of Nature and Science, UNITED STATES

## Abstract

The Early Cretaceous (?Berriasian-Barremian) Teete vertebrate locality in Western Yakutia, East Siberia, Russia, has produced mammal remains that are attributed to three taxa: Eleutherodontidae indet. cf. *Sineleutherus* sp. (Haramiyida; an upper molariform tooth), *Khorotherium yakutensis* gen. et sp. nov. (Tegotheriidae, Docodonta; maxillary fragment with three molariform teeth and dentary fragment with one molariform tooth), and *Sangarotherium aquilonium* gen. et sp. nov. (Eutriconodonta incertae sedis; dentary fragment with one erupted molariform tooth and one tooth in crypt). This is the second occurrence of Mesozoic mammals in high latitudes (paleolatitude estimate N 63–70°) of the Northern Hemisphere. In spite of the presumed Early Cretaceous age based on freshwater mollusks, the Teete mammal assemblage has a distinctive Jurassic appearance, being most similar to the Middle-Late Jurassic mammal assemblages known from Siberia, Russia and Xinjiang, China. The smooth transition from Jurassic to Cretaceous biota in Northern Asia is best explained by stable environmental conditions.

## Introduction

**Fig 1:**
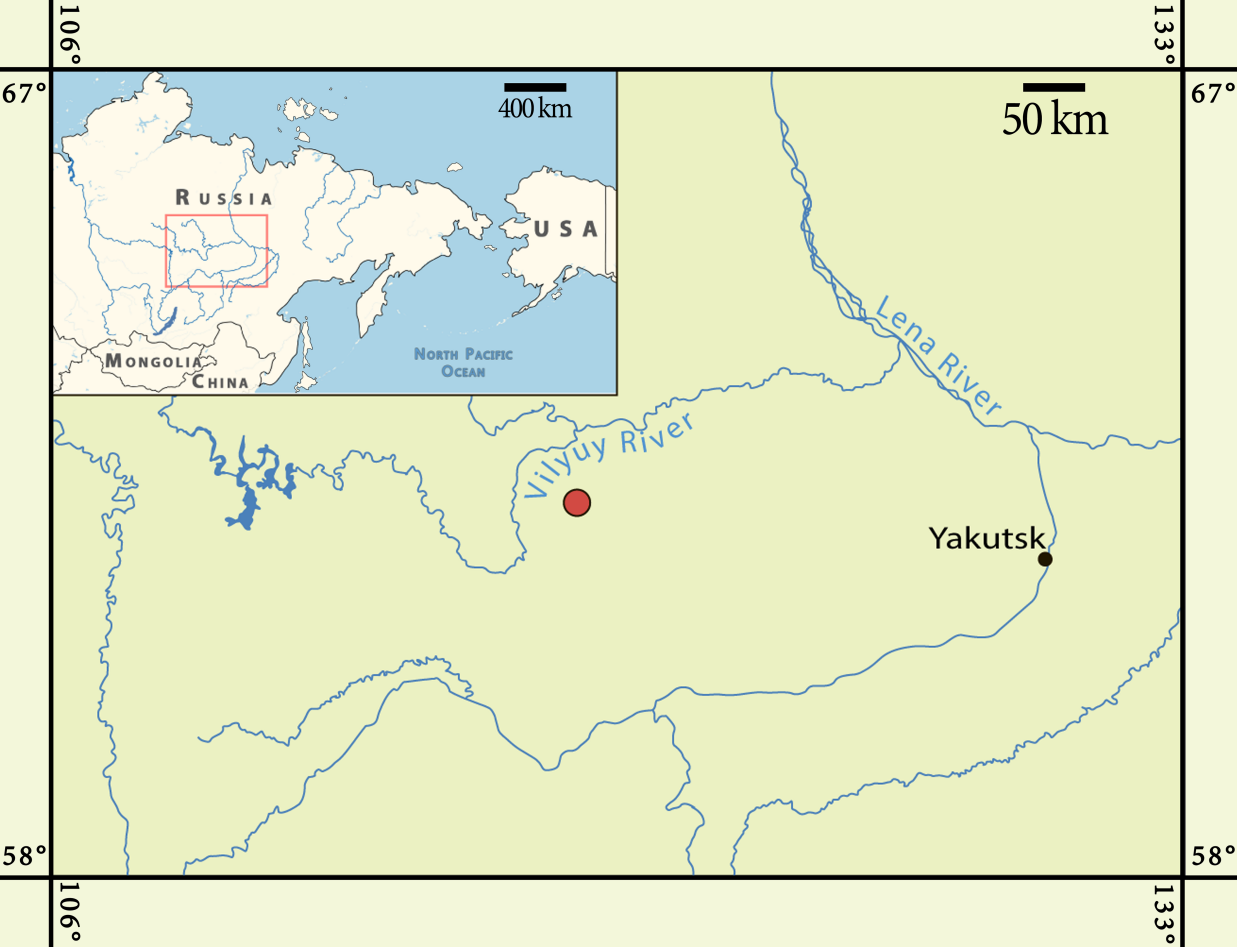
Maps showing the geographic position of the Teete locality (red dot).

The recent decades witnessed a tremendous progress in the study of Mesozoic mammals [1, 2]. However, almost all known Mesozoic mammal localities are confined to low latitude areas. High latitude Mesozoic mammals have been known previously only from one Late Cretaceous (Maastrichtian) site in Alaska, USA and two Early Cretaceous sites in Victoria, Australia [1, 3, 4]. Here we report the second occurrence of Mesozoic mammals in high latitudes of the Northern Hemisphere. This is the Teete (= Kempendyay) locality in northern Yakutia, Russia (Fig 1), which has become known previously by finding of polar dinosaurs [3, 4].

The first dinosaur bones at Teete, including humerus, scapula, dorsal and caudal vertebrae, and ribs, were found by the geologist V.F. Filatov during the Yakutian Geological Expedition in 1960 and subsequently were preliminarily attributed to an ankylosaur [5, 6]. At the time the locality was called Kempendyay after the Kempendyay River, a tributary of the Vilyuy River. In 1988 the locality was visited by a joint expedition of the Moscow Paleontological Institute and the Yakutsk Institute of Geology. This expedition collected isolated teeth of sauropods, theropods, and stegosaurs, as well as bones of small reptiles, possible choristoderes, and pterosaurs [7, 8]. The locality was renamed Teete after the Teete River, a tributary of Botomoiu River. In 2002–2012 the locality was studied by a team from the Institute of Diamond and Precious Metals Geology in Yakutsk led by P. Kolosov [9]. This work led to the discovery of new faunal elements (amphibians, lizards), including the first named vertebrate taxon from the Teete locality–the tritylodontid synapsid *Xenocretosuchus kolossovi* Lopatin and Agadjanian, 2008, which recently was attributed to the genus *Stereognathus* Charlesworth, 1855 [9–12]. Remains of choristoderes collected at the same time were identified as Choristodera indet. [13, 14].

**Fig 2:**
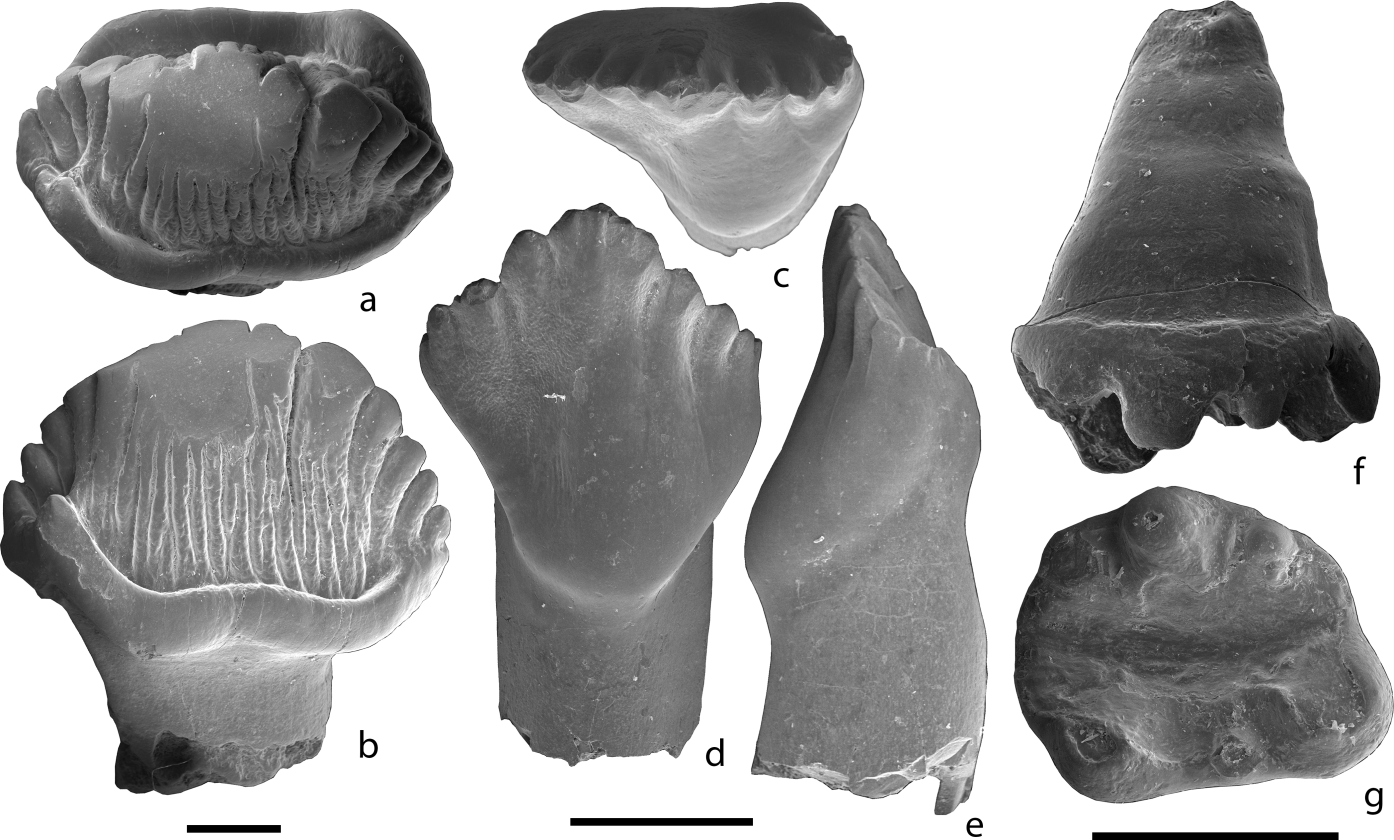
Teeth of Stegosauria indet. **(a, b), Ornithischia indet. (c-e), and Eleutherodontidae indet. cf. *Sineleutherus* sp. (f, g).** a, b, ZIN PH 1/246, in occlusal (a) and labial or lingual (b) views. c-e, ZIN PH 2/246, in occlusal (c), lingual (d), and mesial or distal (e) views. f, g, PIN 5614/4, left upper molariform tooth, in labial (f) and occlusal (g) views. Scale bars equal 1 mm.

In 2017 P. Skutschas, R. Schellhorn, and D. Vitenko conducted new field work at the Teete locality. They screen-washed 500 kg of fossiliferous matrix and recovered numerous vertebrate microfossils, including remains of fishes, salamanders, turtles, choristoderes, lizards, dinosaurs (Fig 2A–2E), tritylodontids, and mammals. Among the mammalian remains collected in 2017 at Teete, there are three toothed jaw fragments (PIN 5614/1-3) referred to a new taxon of tegotheriid docodontan and a new taxon of eutriconodontan, one undetermined docodontan dentary fragment with deciduous premolars (PIN 5614/7), two large double-rooted canines (PIN 5614/8-9), an isolated upper molariform tooth of an eleutherodontid haramiyidan cf. *Sineleutherus* sp. (PIN 5614/4; Fig 2F and 2G), an edentulous dentary fragment (PIN 5614/6) with alveoli for incisors, large double rooted canine (but distinctly smaller than isolated canines), and two premolars, a proximal fragment of robust ulna (PIN 5614/5), and several smaller edentulous dentary and maxilla fragments. The large isolated canines and ulna fragment may belong to a docodontan.

**Fig 3:**
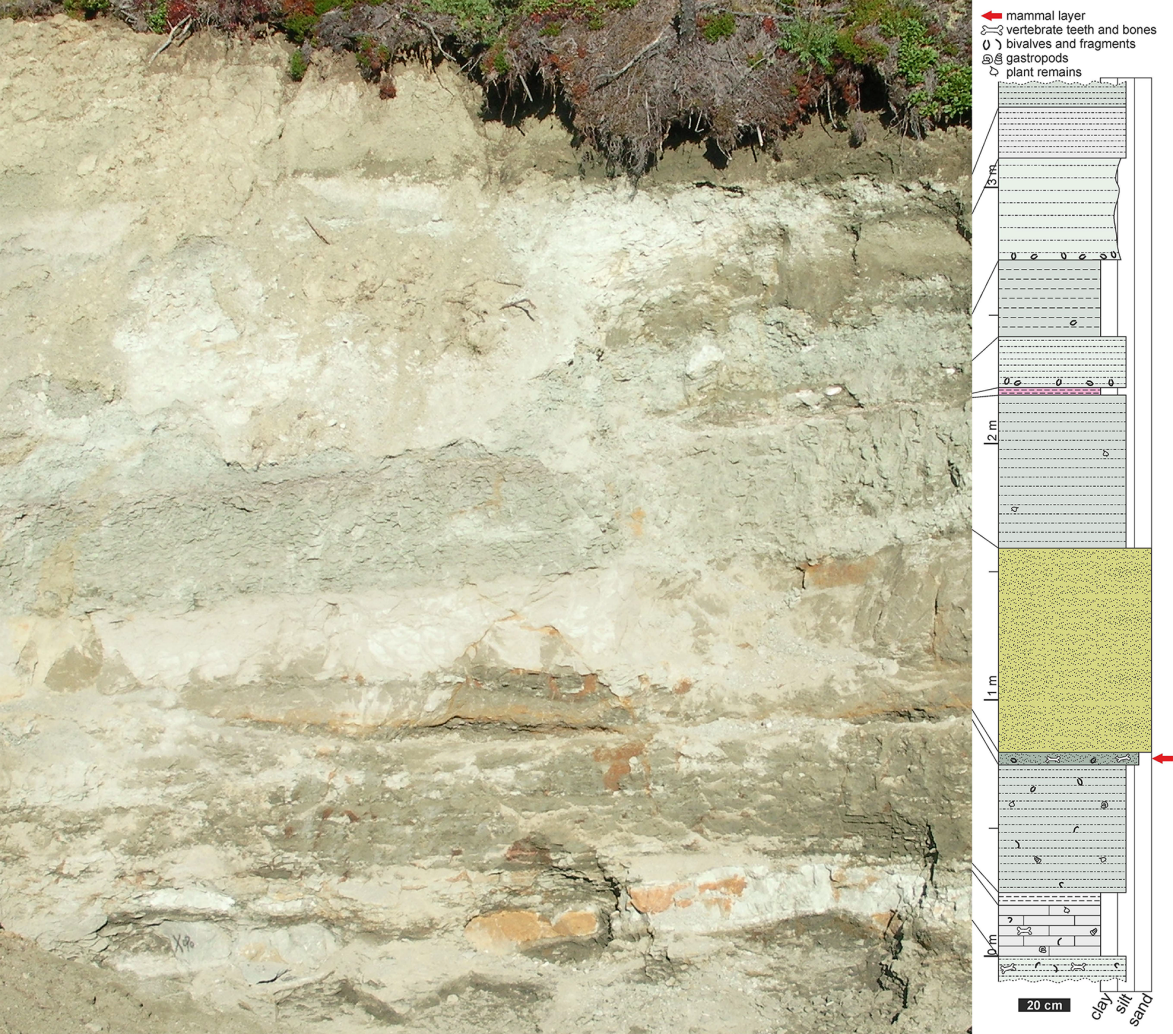
Photograph of the Teete microvertebrate locality (left) and its geological section (right).

**Fig 4:**
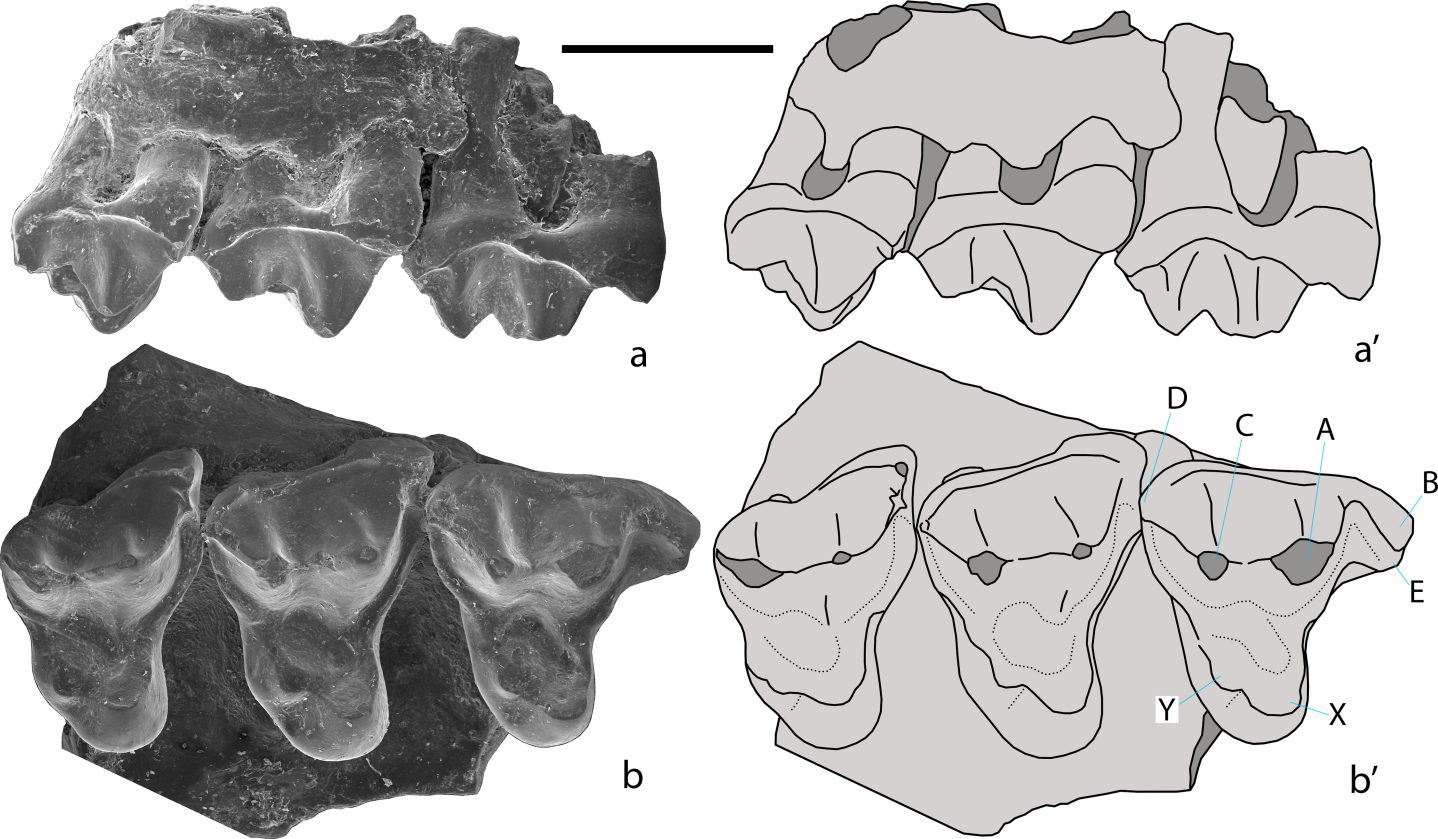
***Khorotherium yakutensis* gen. et sp. nov., PIN 5614/1, right maxilla fragment with the three last molariform teeth (holotype; a, b, SEM micrographs; a’, b’, explanatory drawings)**. Teete, Yakutia, Russia; Sangar Series, Lower Cretaceous. a, labial view. b, occlusal view. A, B, C, D, E, X, and Y–cusps of an upper molariform tooth. Scale bar equals 1 mm.

**Fig 5:**
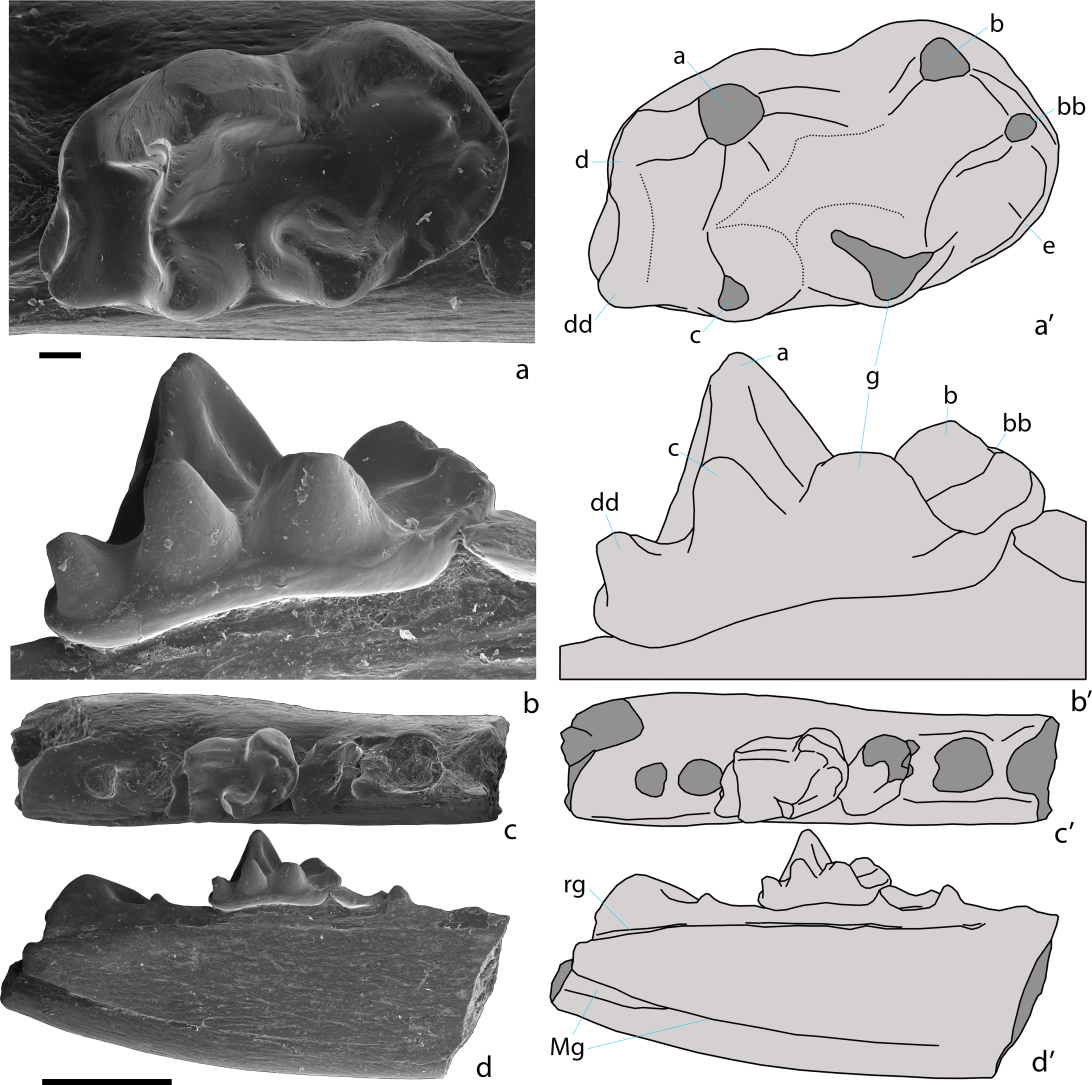
***Khorotherium yakutensis* gen. et sp. nov., PIN 5614/2, left dentary fragment with the penultimate molariform tooth, a distal fragment of the antepenultimate molariform tooth, and alveoli for the ultimate and more anterior teeth (a-d, SEM micrographs; a’-d’, explanatory drawings).** Teete, Yakutia, Russia; Sangar Series, Lower Cretaceous. a, b, penultimate molariform tooth in occlusal (a) and lingual (b) views. c, d, dentary fragment in occlusal (c) and lingual (d) views. Abbreviations: Mg, Meckel's groove; rg, replacement groove. a, b, bb, c, d, dd, e, and g–cusps of a lower molariform tooth. Scale bars equal 0.1 mm for a, b, and 1 mm for c, d.

For docodontans we use dental terminology proposed by Butler [15] with subsequent modifications [16, 17]. For eutriconodontans we use the conventional cusp numbering [1]. The cusps are labelled on Figs 3–5.

## Nomenclatural acts

The electronic edition of this article conforms to the requirements of the amended International Code of Zoological Nomenclature, and hence the new names contained herein are available under that Code from the electronic edition of this article. This published work and the nomenclatural acts it contains have been registered in ZooBank, the online registration system for the ICZN. The ZooBank LSIDs (Life Science Identifiers) can be resolved and the associated information viewed through any standard web browser by appending the LSID to the prefix "http://zoobank.org/". The LSID for this publication is: urn:lsid:zoobank.org:pub: C0258A19-FB74-4E4E-A530-B5CF6809FA66. The electronic edition of this work was published in a journal with an ISSN, and has been archived and is available from the following digital repositories: PubMed Central and LOCKSS.

## Geological setting

In the Middle Jurassic–Early Cretaceous (Callovian-Albian) the Vilyuy River Basin was part of the Vilyuy syneclise, a depressed region of the Siberian Platform. In the Vilyuy syneclise a thick sequence of predominantly coal-bearing, but laterally highly variable deposits was formed during this time [18]. These deposits comprise sandstones, siltstones, and clays, formally designated the Sangar Series. The total thickness of the Sangar Series is about 1500 m. The Sangar Series is divided from oldest to youngest into the Batylykh, Eksenyakh, and Khatyryk formations [19]. The Teete locality is located in South-West part of the Vilyuy syneclise at the junction of Suntar uplift and Kempendyay Depression. Stratigraphically it is placed within the Batylykh Formation, the lowest formation of the Sangar Series.

The base of the section at the Teete locality is comprised of green silts with randomly dispersed shell fragments of bivalves and gastropods (Fig 3). Within this layer of unknown thickness dinosaur teeth as well as larger rib and pelvic fragments (up to 20 cm long) can be found. According to Kolosov et al. 2009 this layer is comprised of psammitic tuffites and a bonebearing bed [9]. Overlying the outcrop, is a dark grey micritic limestone with small black coalified plant remains up to 1 cm in length. This 15 to 20 cm thick limestone also contains remains of bivalves and gastropods, and also bone fragments. The base of the limestone is light grey in color. In general the limestone seems to be broken up in lenses probably due to cryogenic weathering in the permafrost soil. This limestone is around meter 15 in the Kolosov et al. 2009 section [9]. A 5 cm thin layer of white to light grey marly clays lies upon the limestone. Overlying this white layer is an up to 50 cm thick layer of green to grey silts with randomly dispersed shell fragments up to 1 cm in length. There are also complete bivalve and gastropod shells within this layer. Large bivalves (up to 7 cm in length) are present only in the upper part of this layer. The "mammal layer" that produced the mammal teeth describe here, also produced a few fish scales, dinosaur teeth, and bone fragments among others. This fossil rich layer is 2 to 5 cm thick and comprised of clay to sand. Above this layer is an up to 80 cm thick layer of quartz sand, the coarsest sediment within the section. This yellow to greenish-grey layer is hardened but weakly lithified. Neither sedimentary structures nor fossil remains can be observed in this layer, but light and dark mica are visible. Up to 60 cm of green to grey silt are overlie the hard sand layer. These silts with coalified plant remains are somewhat more compactified and lighter in color at the base of this layer. The most eye-catching layer is a thin (2–4 cm) layer of pinkish marly clays. The overlying 20 cm thick green silt layer contains sporadically distributed large (up to 7 cm in length) bivalve shells. Above this layer, the 30 cm thick grey to greenish-grey layer of clay also contains some of these large bivalves and is thinning laterally to a thickness of 5 cm or less. An up to 40 cm thick layer of greenish fine silt (and marly clay at the base) show up to 7 cm long bivalves in-situ at the base of the layer. The top of the section is silty. The light grey 20 cm thick layer is thinning laterally and disappearing. The top-layer of the section and the outcrop is comprised of silts and is of unknown thickness.

In Yakutia the climatic and environmental conditions during the Early Cretaceous were essentially the same as in the Late Jurassic [20]. The faunistic and floristic changes from the Late Jurassic to the Early Cretaceous were gradual in that region, which makes the age determination of the Teete locality difficult [9]. Within the Sangar Series, there are two levels with rich assemblages of freshwater molluscs, one similar to that from the Valanginian of Transbaikalia, and the other to the assemblages from the Hauterivian-Barremian of Transbaikalia and Mongolia [19, 21, 22]. The fresh-water molluscs collected from the fossiliferous level at the Teete locality include taxa corresponding to the Valanginian fauna. The Batylykh Formation in the lower reaches of the Vilyuy River was considered to be Neocomian based on plant macrofossils [23]. The pollen assemblage collected from the Teete sections contains typical Neocomian (Lower Cretaceous) taxa with some Late Jurassic elements [9]. Currently the best age estimate for the Teete vertebrate locality is the lower part of the Lower Cretaceous (Berriasian-Barremian).

## Systematic paleontology

**Mammalia** Linnaeus, 1758 [24]

**Docodonta** Kretzoi, 1946 [25]

**Tegotheriidae** Tatarinov, 1994 [26]

Genus ***Khorotherium*** gen. nov.

urn:lsid:zoobank.org:act:33E9EFA8-BAA5-481D-993D-51AE3109B7F5

### Type species

*Khorotherium yakutensis* sp. nov.

### Diagnosis

Referred to Tegotheriidae [17] based on the absence of A-X crest, presence of cusp bb, presence of a large pseudotalonid basin bordered by crests a-b, b-bb, bb-g, and a-g, and absence of crest b-g on the lower molariform teeth. Similar to *Tegotherium* Tatarinov, 1994 from the Late Jurassic of Mongolia and Xinjiang, China [26, 27] by lack of the mesio-lingual cusp Z; differs from *Tegotherium* by a mesial cingulum not extending on the lingual part of the crown of the upper molariform teeth, by lack of the crest extending from cusp X towards the base of cusp A, and by a thinner Meckel’s groove. Differs from *Hutegotherium* Averianov et al., 2010 from the Middle Jurassic of West Siberia, Russia by absence of an ectoflexus on the anterior upper molariform teeth, by a much smaller cusp B, by absence of cusps Z and Y1, by mesial and distal cingula that do not extend onto the lingual part of the crown of the upper molariform teeth, by a cusp g that is larger than cusp c, and by an incomplete crest a-g. Differs from *Sibirotherium* Maschenko et al., 2003 from the Early Cretaceous of West Siberia, Russia [28, 29] by absence of an ectoflexus at the anterior upper molariform teeth, by absence of cusp Z, and by an incomplete crest a-g. Differs from *Tashkumyrodon* Martin and Averianov, 2004 from the Middle Jurassic of Kyrgyzstan [30] by strong reduction of cusp d and by the lack of crest c-d. Differs from *Krusatodon* Sigogneau-Russell, 2003 from the Middle Jurassic of Great Britain [31] by an incomplete crest a-g and a larger cusp dd. Differs from all known taxa of Docodonta by presence of a replacement groove.

### Etymology

From Khoro village in Suntar Ulus, Yakutia, the nearest settlement (about 70 km) to the Teete locality and Greek θηρίον (beast).

***Khorotherium yakutensis*** sp. nov.

urn:lsid:zoobank.org:act:E615614E-B2DE-4B44-B603-E1E5602FC142

Figs 4–5

### Holotype

PIN 5614/1, right maxilla fragment with three last molariform teeth.

### Referred specimen

PIN 5614/2, left dentary fragment with penultimate molariform tooth, distal fragment of the antepenultimate molariform tooth, and alveoli for the ultimate and more anterior teeth.

### Type locality and horizon

Teete, Yakutia, Russia. Batylykh Formation, Sangar Series, Lower Cretaceous.

### Etymology

From Yakutia where the fossils were discovered.

### Description

The anterior root of the maxillary zygomatic process starts between the first and second molariform teeth. The posterior root of this process is at the distal margin of the last molariform tooth.

The three molariform teeth have a similar morphology. The positional variation concerns the size and direction of the mesio-labial lobe, which is very large and mesially directed on the first tooth, and small and mesio-labially directed in the other teeth. The prominent mesio-labial lobe in the first tooth suggests that it is the first molariform. The crowns of the upper molariforms are triangular in shape. Their labial part is dominated by a large mesio-labial cusp A. The disto-labial cusp C is distinctly lower and about three times smaller than cusp A. The height of cusp C markedly decreases in size from the first to the last molariform tooth while the height of cusp A is more constant. The apex of cusp C is placed disto-lingually to the apex of cusp A. There are sharp ridges between cusps A and C. On the first molariform tooth a similar sharp crest connects cusp A and a small cuspule on the labial cingulum, which is widely separated from cusp B. On the second molariform tooth this crest is connecting cusp A with the strongly reduced cusp B. On the third molariform tooth this crest is between cusps A and E. The distal crest connects cusp C and the disto-labial cingulum in the first and second molariform teeth. On the last molariform tooth this crest is absent and the disto-labial cingulum is reduced. The labial side of the crown is almost straight on the first and second molariform teeth, without an ectoflexus. A very shallow ectoflexus is present on the last molariform tooth. The ectocingulum is distinct, thinner in the middle part of the crown. On the first molariform tooth there is a prominent, mesially directing mesio-labial lobe containing the mesial stylar cusps B and E. Cusp B is relatively large. The cusp E is somewhat smaller and almost completely worn. The mesio-labial lobe is greatly reduced, with much smaller cusps B and E, and directed more labially in the second and third molariform teeth. The cingulum surrounds the labial part of the crown, except the disto-labial corner of cusp C on the last molariform tooth. The cingulum does not extend onto the lingual part of the crown. The lingual part of the crown is half as long as the labial part. It is proportionally largest on the second molariform tooth. The lingual part of the crown bears two cusps and has a relatively large talon basin. The mesio-lingual cusp X is the largest lingual cusp. It is placed lingually to the other cusps. Cusp X is largest on the second molariform tooth and smallest on the third molariform tooth. The disto-lingual cusp Y is less than half the size of cusp X and placed on the crest connecting cusp X with the distal cingulum. The mesial crest connects cusp X with the mesial cingulum.

The preserved posterior portion of the mandibular body deepens at the base of the coronoid process. The lingual side of the dentary is flat. Meckel's groove is wide posteriorly, but considerably tapers anteriorly and becomes linear below the molariform teeth. It terminates between the ante-penultimate and ultimate molariform teeth. On the lingual side along the alveolar border there is a distinct replacement groove, which deflects ventrally posterior to the last molariform alveolus end extends to the preserved end of the dentary fragment. The ventral border of the dentary is convex.

Judging from the alveoli, the antepenultimate tooth was about 1.5 times larger than the ultimate tooth. The preserved penultimate molariform tooth is intermediate in size between these teeth. The preserved distal part of the ante-penultimate tooth is similar in structure with that part of the penultimate tooth but distinctly larger. The crown of the penultimate tooth is dominated by a large main cusp a, occupying the center and distal part of the labial side of the crown. The mesio-labial cusp b is less than half the size of cusp a and is well separated from the latter. Cusp b is positioned on the depressed part of the crown that exaggerates the height difference between the two cusps. Disto-lingual cusp c is distinctly smaller than mesio-lingual cusp g. Cusp c is placed somewhat distally to cusp a and is connected with the apex of the latter cusp by a strong transverse ridge a-c. Cusp g is located at the level between cusps a and b. The pseudotalonid is bordered by crests a-b, b-bb, bb-g, and incomplete crest a-g. Crest a-g is interrupted in the middle and the pseudotalonid basin is connected with the basin between the main cusps a, c, and g. The lingual part of this crest, extending from the apex of cusp g, is robust and more pronounced than the other part of the crest. Parallel to the crest bb-g there is a prominent mesio-lingual shelf (cusp e). A short ridge extends from the apex of cusp g into this shelf. The distal part of the crown is short and consists of depressions between the transverse crests a-c and d-dd. Cusp dd is prominent and about two times lower than cusp c. Cusp d is hardly individualized. There is a distinct disto-lingual cingulid extending from cusp d in lingual direction. The lingual cingulid between the cusps c and g is very faint.

### Measurements

PIN 5614/1, three upper molariform teeth, measured in anterior-posterior sequence, length = 1.85; 1.75; 1.3; anterior width = 1.9; 2.2; 1.9; posterior width = 2.05; 2.1; 1.6 mm. PIN 5614/2, lower molariform tooth, length = 1.3; width = 0.8 mm.

### Comments

A groove for the replacement dental lamina (Crompton’s groove), which is usually present in non-mammalian cynodonts with ongoing postcanine replacement, is often found on the mandibles of Morganucodonta [32–36]. Among docodontans the replacement groove was documented previously only for an edentulous dentary fragment (PIN 5087/1) from the Middle Jurassic Itat Formation of West Siberia, Russia [37].

A right dentary fragment (PIN 5614/7) with two deciduous premolars (dp?3–4) is in size similar to PIN 5614/2 and may belong to *Khorotherium yakutensis* sp. nov.

**Eutriconodonta** Kermack et al., 1973 [38]

**Eutriconodonta** incertae sedis

Genus ***Sangarotherium*** gen. nov.

urn:lsid:zoobank.org:act:44BB9EBC-11EA-4DCF-A278-4E6038F25906

### Type species

*Sangarotherium aquilonium* sp. nov.

### Diagnosis

Differs from stem mammals with ‘triconodont’ dental pattern (Morganucodonta Kermack et al., 1973 [38] by lack of a lingual cingulum and cingulid cusps, as well as an unreduced cusp b. Differs from Amphilestidae Osborn, 1888 [39] by a distinctly lower cusp a, approximating the height of the side cusps, by cusps b and c that are of unequal height, and by the lack of interlock of the lower molariform teeth with individualized cusps e and f. Differs from Gobiconodontidae Chow and Rich, 1984 [40] by a cusp b that is larger than cusp c, by the lack of a lingual cingulid and by interlocking lower molariform teeth. Differs from Triconodontidae Marsh, 1887 [41] by the unequal height of the lower molariform cusps, by an unreduced cusp e, and by the lack of a tongue-in-groove interlocking mechanism at the lower molariform teeth.

### Etymology

From Sangar Series, a stratigraphic unit where the fossil was found, and Greek, θηρίον (beast).

***Sangarotherium aquilonium*** sp. nov.

urn:lsid:zoobank.org:act:AA230F83-AD85-47E1-B323-B95A5655EEE1

Fig 6

**Fig 6:**
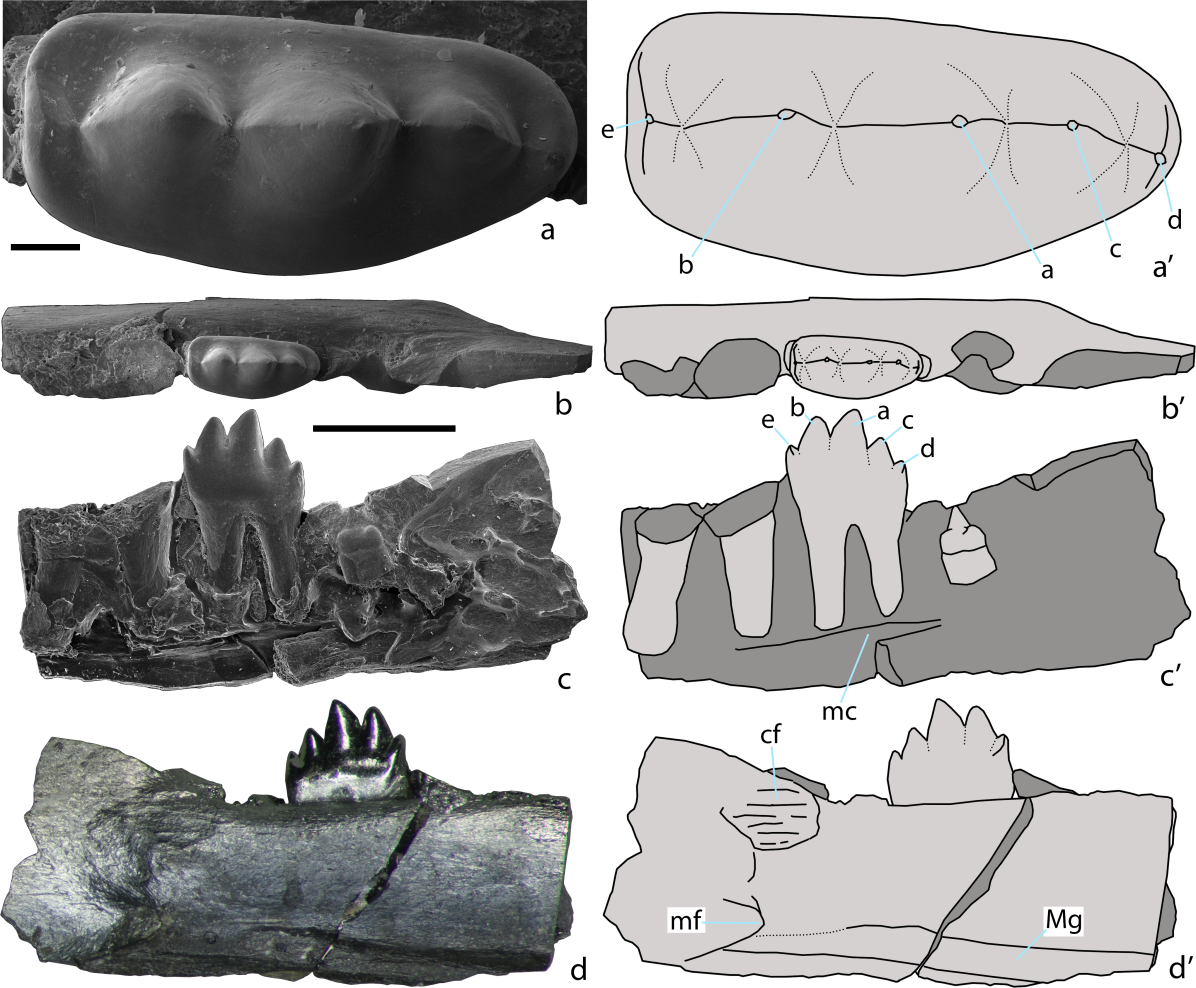
***Sangarotherium aquilonium* gen. et sp. nov., PIN 5614/3, left dentary fragment with roots of the antepenultimate molariform tooth, the penultimate molariform tooth, and the ultimate molariform tooth in crypt (holotype; a-c, SEM micrographs; d, light photograph; a’-d’, explanatory drawings).** Teete, Yakutia, Russia; Sangar Series, Lower Cretaceous. a, penultimate molariform tooth in occlusal view. b-d, dentary fragment in occlusal (b), labial (c), and lingual (d) views. Abbreviations: cf, coronoid facet; mc, mandibular canal; mf, mandibular foramen; Mg, Meckel's groove. a, b, c, d, e–cusps of a lower molariform tooth. Scale bars equal 0.1 mm for a and 1 mm for b-d.

### Holotype

PIN 5614/3, left dentary fragment with roots of the antepenultimate molariform tooth, the penulatimate molariform tooth, and the cryptic ultimate molariform tooth.

### Type locality and horizon

Teete, Yakutia, Russia. Batylykh Formation, Sangar Series, Lower Cretaceous.

### Etymology

From Latin aquilōnius (northern).

### Description

The labial side of the dentary is broken and reveals the three last molariform teeth: the roots of the antepenultimate tooth, the complete penultimate tooth, and the complete unerupted ultimate tooth preserved in a crypt. The mandibular canal is placed in the ventral third of the mandibular body. A small part of the ventral margin of the dentary is missing. In spite of this, it is evident that the dentary was gracile in contrast to the robust dentary in most eutriconodontan taxa [1]. The dentary depth is twice the height of the preserved molariform tooth which was likely not the highest in the tooth series. On the lingual side at the base of the mostly missing coronoid process there is a well-defined rugose area, likely an attachment area for the coronoid bone. The mandibular foramen is placed at the level of the anterior end of the coronoid process. Meckel's groove is relatively wide dorso-ventrally and has a slightly undulating pattern. It is almost constant in width along the entire preserved dentary fragment. The groove is shallow in the vicinity of the mandibular foramen but deepens anteriorly.

The ante-penultimate molariform tooth is about one third longer than the preserved penultimate tooth. Its distal root is larger than the medial root in contrast to the penultimate molariform tooth.

The penultimate molariform tooth has a typical ‘triconodont’ molar pattern with five linearly arranged cusps. The relative height of the cusps is the following: a > b > c > e > d. The height differential between the main cusp a and the adjacent cusps is relatively low, less than on the posterior molariform teeth of amphilestids and gobiconodontids that have a low cusp a. Main cusp a is somewhat asymmetrical, with an oblique mesial shearing margin and a more vertical distal margin. Cusps b and c are more symmetrical. The marginal cusps e and d are quite distinct and connected by longitudinal ridges with the central cusps. The crown is relatively wide labio-lingually withthe labial side distinctly convex and the lingual side almost straight. There is no cingulid on either the labial or the lingual side. Short arms of the mesial and distal cingulid are connected with cusps e and d, respectively. The mesial side is straight without any sign of interlocking with the previous molariform tooth. The crown is mesio-distally wider than the roots, extending mesially and distally beyond the crown-root junction.

The ultimate molariform tooth, preserved in its crypt, is single-rooted and has a crown with a single conical cusp having a flattened labial side.

### Measurements

PIN 5614/3, penultimate lower premolar, length = 1.1; width = 0.5 mm.

### Comments

A coronoid facet for *Gobiconodon ostromi* Jenkins and Schaff, 1988 from the Early Cretaceous of Montana, USA was reported in ref. [42]. However, there is no clear coronoid facet in the *Gobiconodon* species from the Early Cretaceous of Mongolia [43, 44], although some rugosity can be present at this place. A slit at the base of the coronoid process in a juvenile dentary of *Hangjinia chowi* Godefroit and Guo, 1999 from the Early Cretaceous of Inner Mongolia, China, was interpreted as a coronoid facet in ref. [45]. There is no coronoid bone present in a well preserved skull of *Liaoconodon hui* Meng et al. 2011 from the Early Cretaceous of Liaoning, China [46]. The coronoid facet is absent in other eutriconodontans [1]. The well-defined rugose coronoid facet in *Sangarotherium* gen. nov. is the most plesiomorphic condition of character known for the Eutriconodonta.

The size of Meckel's groove in PIN 5614/3 suggests that it was likely housing an ossified Meckel's cartilage [46–49].

## Discussion

The paleolatitude of the Teete (‘Kempendyay’) locality was estimated as N 63–70° [3, 4]. This is close to the paleolatitude estimate (N 70–85°) for the early Maastrichtian vertebrate locality at the Colville River in Alaska, USA (Prince Creek Formation) [50, 51]. The metatherians, and eutherians from the Colville River vertebrate assemblage resemble those from the Maastrichtian sites in Wyoming, Montana (USA) and Alberta (Canada) but are different on the species level, while the multituberculates are represented by discrete taxa [50, 52]. The dinosaurs of the Prince Creek Formation are represented by theropod teeth, similar to the southern taxa, while the more complete specimens belong to endemic taxa of Tyrannosauridae, Pachycephalosauria, and Ceratopsidae [53–58]. The Colville River vertebrate assemblage is remarkable in the lack of amphibians and non-dinosaurian sauropsids, such as turtles, squamates, and crocodyliforms. This was interpreted as evidence for a climatic control of the distribution of the animals with only endothermic tetrapods being able to live in such high latitudes [50]. The Teete vertebrate assemblage is only the second known high latitude Mesozoic vertebrate fauna in the Northern Hemisphere containing skeletal remains of mammals. Mammalian ichnofossils are known from a high-latitude locality within the Campanian Wapiti Formation in Canada [59]. The Teete vertebrate assemblage contains both endothermic, or presumably endothermic tetrapods (dinosaurs, tritylodontids, and mammals) and ectothermic tetrapods (salamanders and lizards). However, the remains of turtles, which are the most common tetrapod elements in the majority of the Mesozoic vertebrate assemblages in lower latitudes, are extremely rare in the Teete locality. In the Jurassic and Early Cretaceous turtles so far have not been recorded from areas north of N 60° paleolatitude [60]. A few remains from the Teete locality are the first record of turtle in such high latitudes in Asia. The remains of crocodyliforms, another tetrapod group that is in more southern Late Mesozoic vertebrate faunas, have not been recorded so far from there. In the Cretaceous crocodyliforms did not live in regions with a minimum mean annual temperature below 14°C [61]. However, during the Eocene thermal maximum, both turtles and crocodyliforms lived in North America above the Arctic Circle and may have survived minimum mean annual temperatures below 14°C [62–64]. The presence of salamanders, turtles, and lizards in the Teete vertebrate assemblage, in contrast with the Colville River vertebrate assemblage, may be explained by a somewhat more southern position of the Yakutian locality and warmer climatic conditions.

According to the hypothesis by Amiot et al. [65], there was a climatic barrier in Asia during the Early Cretaceous that separated the distribution of choristoderes (which were more tolerant of cold temperatures) and thermophilic crocodyliforms (see also [66]). This supposed tolerance of cold temperatures may explain the presence of choristoderes in the Teete locality [14].

The currently known mammal assemblage from the Teete locality includes an eleutherodontid haramiyidan, a docodontan, and a eutriconodontan. It is similar in composition with a much better-known mammal assemblage from the Middle Jurassic (Bathonian) Itat Formation at the Berezovsk coal mine in Krasnoyarsk Territory, Russia (see [67] for recent review of the fauna). The Berezovsk mammal assemblage is dominated by eleutherodontid haramiyidans and docodontans. Dryolestids, stem therians, and eutriconodontans are less common. A similar composition is present in Jurassic mammal assemblages known from northwestern and northeastern regions of China. In the Junggar Basin, Xinjiang, China, mammals are known from the Upper Jurassic (Oxfordian) Qigu Formation [27, 68, 69]. This mammal assemblage is also dominated by eleutherodontid haramiyidans and docodontans, while remains of stem therians are rare. A more diverse mammal fauna is known from the Middle-Upper Jurassic Lagerstätten assemblages widely distributed in Nei Mongol, Liaoning, and Hebei, North-East China [70]. The vertebrate assemblages from these Lagerstätten, collectively termed the Daohugou Biota, include diverse haramiyidans, docodontans, eutriconodontans, pseudotribosphenic mammals, and stem therians. In contrast, the mammal assemblage from the Early Cretaceous (Barremian-Aptian) Jehol Biota of northeastern China, is dominated by eutriconodontans and symmetrodontans, and includes also multituberculates and stem placentals [71, 72]. The mammal assemblages from the Lower Cretaceous (Barremian-?Aptian) Ilek Formation, widely distributed in western Siberia (Kemerovo Province and Krasnoyarsk Territory, Russia) have a somewhat intermediate composition between the mammal assemblages from Daohugou and Jehol Biotas. They are dominated by symmetrodontans and eutriconodontans; docodontans are still present and multituberculates are very rare [73–75]. As presently known, the Teete mammal assemblage is essentially similar with the Jurassic Laurasian mammal assemblages in the presence of haramiyidans and docodontans. In the Ilek mammal assemblage docodontans are present, but there are no haramiyidans. The Teete mammal assemblage is clearly different from the Early Cretaceous mammal assemblages of Asia by the lack of symmetrodontans. The only eutriconodontan present in the fauna, *Sangarotherium aquilonium* gen. et sp. nov., is clearly different from the gobiconodontids which were among the dominant groups of early Cretaceous mammals in Asia. Thus, in spite of the supposed Lower Cretaceous age of the fossiliferous sediments in the Teete locality, the mammal assemblage from there has a distinctive Jurassic appearance. This may be explained by stable environmental conditions in North Asia and gradual transition between the Jurassic and Cretaceous biotas, in contrast with the environmental perturbations that occurred in other regions at the Jurassic/Cretaceous boundary [76].

## Abbreviations

PIN: Borissiak Paleontological Institute, Russian Academy of Sciences, Moscow
ZIN PH: Paleoherpetological collection, Zoological Institute, Russian Academy of Sciences, Saint Petersburg
